# Immune function during pregnancy varies between ecologically distinct populations

**DOI:** 10.1093/emph/eoaa022

**Published:** 2020-07-03

**Authors:** Carmen Hové, Benjamin C Trumble, Amy S Anderson, Jonathan Stieglitz, Hillard Kaplan, Michael D Gurven, Aaron D Blackwell

**Affiliations:** e1 Department of Anthropology, University of California Santa Barbara, Santa Barbara, CA 93106, USA; e2 School of Human Evolution and Social Change, Center for Evolution and Medicine, Arizona State University, Tempe, AZ 85287, USA; e3 Institute for Advanced Study in Toulouse, Toulouse 31015, France; e4 Economic Science Institute, Chapman University, Orange, CA 92866, USA; e5 Department of Anthropology, Washington State University, Pullman, WA 99164, USA

**Keywords:** reproductive ecology, fetal tolerance, ecological immunology, Tsimane

## Abstract

**Background and objectives:**

Among placental mammals, females undergo immunological shifts during pregnancy to accommodate the fetus (i.e. fetal tolerance). Fetal tolerance has primarily been characterized within post-industrial populations experiencing evolutionarily novel conditions (e.g. reduced pathogen exposure), which may shape maternal response to fetal antigens. This study investigates how ecological conditions affect maternal immune status during pregnancy by comparing the direction and magnitude of immunological changes associated with each trimester among the Tsimane (a subsistence population subjected to high pathogen load) and women in the USA.

**Methodology:**

Data from the Tsimane Health and Life History Project (*N* = 935) and the National Health and Nutrition Examination Survey (*N* = 1395) were used to estimate population-specific effects of trimester on differential leukocyte count and C-reactive protein (CRP), a marker of systemic inflammation.

**Results:**

In both populations, pregnancy was associated with increased neutrophil prevalence, reduced lymphocyte and eosinophil count and elevated CRP. Compared to their US counterparts, pregnant Tsimane women exhibited elevated lymphocyte and eosinophil counts, fewer neutrophils and monocytes and lower CRP. Total leukocyte count remained high and unchanged among pregnant Tsimane women while pregnant US women exhibited substantially elevated counts, resulting in overlapping leukocyte prevalence among all third-trimester individuals.

**Conclusions and implications:**

Our findings indicate that ecological conditions shape non-pregnant immune baselines and the magnitude of immunological shifts during pregnancy via developmental constraints and current trade-offs. Future research should investigate how such flexibility impacts maternal health and disease susceptibility, particularly the degree to which chronic pathogen exposure might dampen inflammatory response to fetal antigens.

**Lay Summary:**

This study compares immunological changes associated with pregnancy between the Tsimane (an Amazonian subsistence population) and individuals in the USA. Results suggest that while pregnancy enhances non-specific defenses and dampens both antigen-specific immunity and parasite/allergy response, ecological conditions strongly influence immune baselines and the magnitude of shifts during gestation.

## INTRODUCTION

Among placental mammals, female reproduction requires hosting genetically non-identical offspring throughout gestation without rejecting fetal tissue—a phenomenon termed fetal tolerance [1]. In humans (as well as chimpanzees and gorillas), placentation is particularly invasive [2, 3] and fetal tolerance requires systemic changes in maternal immune function that alter susceptibility to certain infections and autoimmune disorders [4–7]. To date, fetal tolerance has been studied primarily in high-income post-industrial countries, such as the USA and the UK, where epidemiological transitions over the past several centuries have largely curbed exposure to numerous parasites, pathogens and microbes otherwise abundant throughout human history prior to industrialization [8, 9]. Such microbial deprivation lowers overall infectious disease mortality but provides fewer opportunities for immune calibration, paving the way for immunological hypersensitivity to non-pathogenic stimuli and development of myriad chronic inflammatory disorders (e.g. allergy, rheumatoid arthritis) common across industrialized populations [10]. The potential effects of impaired immune calibration on maternal responses to fetal antigens likely interact with other common and evolutionarily novel aspects of post-industrial societies, such as elevated rates of obesity (a chronic inflammatory state) [11] and low parity (which results in less ‘exposure’ to fetal antigens over time). Considering the current characterization of fetal tolerance is primarily derived from populations experiencing such ecological conditions, understanding of how fetal tolerance is shaped by environments more closely resembling those experienced throughout most of human evolution and by many women alive today is limited.

From both an evolutionary perspective and a public health standpoint, there is a pressing need to examine the effect of pregnancy on maternal immunity across diverse environments, particularly those characterized by elevated infection risk. To this end, this study estimates and compares the effect of pregnancy on differential leukocyte count and C-reactive protein (CRP) concentration among Tsimane women, a natural-fertility subsistence population inhabiting a pathogen-rich environment, and women in the USA (Table 1).

**Table 1.** General descriptions of immunological biomarkers used in this study

Total leukocytes	The total number of nucleated immune cells circulating in the blood, also known as WBC count.
Lymphocytes	A heterogenous group of cells identified based on the presence of CD45 receptors. Made up of T, B and Natural Killer cells that together mediate antigen-specific immunity.
Neutrophils	Abundant phagocytic granulocytes that respond to extracellular and fungal infection, prime the adaptive response and promote inflammation.
Monocytes	Phagocytic granulocytes, similar in function to neutrophils but shorter-lived and less prevalent in the blood. Migrate to sites of infection where they differentiate into tissue-specific macrophages.
Eosinophils	Granulocytes involved in parasite response and allergy. Chronically elevated in populations where parasite exposure is high.
Basophils	A rare type of granulocyte involved in inflammation and parasite response.
CRP	Non-specific acute-phase reactant released by the liver in response to inflammation.

### Maternal immune changes during pregnancy

In humans and other mammalian species with haemochorial placentation, gestation results in exposure to both fetal cells [12] and cell-free fetal DNA [13], prompting systemic recalibration of maternal immune function and changes in maternal disease susceptibility [14]. During pregnancy, women are generally more vulnerable to intracellular pathogens [4] but exhibit elevated resistance to extracellular microbes [5]. Pregnancy also exerts contrasting effects on autoimmunity: certain autoimmune diseases, such as rheumatoid arthritis, temporarily resolve during gestation [15] while others worsen [16]. These alterations in disease susceptibility have been linked to quantitative shifts in immune components, though the underlying causal pathways are only partially elucidated. Pregnancy is often described as a state of regulated inflammation [17] and is consistently marked by a rise in the total number of circulating leukocytes [18], primarily due to expansion of neutrophils and monocytes [19]. Neutrophils and monocytes are phagocytic cells that induce inflammatory activation and strengthen resistance to extracellular infections, but are also implicated in the etiology of autoimmune diseases [20] and deleterious inflammation-mediated conditions, such as preterm birth [21]. Total lymphocyte counts are consistently reduced during pregnancy, primarily due to fewer circulating cytotoxic lymphocytes capable of directly recognizing and targeting fetal antigens [22, 23]. Conversely, proliferation of regulatory lymphocytes is heightened [24], further moderating the response to fetal antigens. Eosinophils mediate parasitic and allergic responses and are often reduced among pregnant women [25], though the role of pregnancy-induced eosinopenia is relatively understudied. Taken together, these patterns (based primarily on cross-sectional studies of women in high-income industrialized countries) suggest that pregnancy induces heavier reliance on non-specific defenses and down-regulation of antigen-specific immunity and immune components involved in allergy/parasite response, thereby facilitating fetal tolerance while preserving general pathogen defense.

### Immune function among the Tsimane

The Tsimane inhabit the Bolivian neo-tropics, an environment rich in biodiversity and home to myriad infectious agents. Due to varied and chronic exposure, infections (largely respiratory and gastrointestinal) are the primary drivers of morbidity and mortality among the Tsimane, while allergies, atopy and autoimmune diseases are rare [26]. At any given time, the majority of Tsimane individuals are infected with at least one species of helminth, and over a third are infected with giardia [27]. Furthermore, Tsimane women have restricted access to contraception and the average total fertility rate is nine births per woman [28]. On average, the Tsimane exhibit developmental trajectories in which energetic investment is preferentially shunted into overall immune function, resulting in higher resting metabolic rate and total energy expenditure [29], reduced growth [30] and elevated baseline levels of most immunological parameters compared to Western clinical standards [31]. Average leukocyte counts are higher among the Tsimane, due to more circulating lymphocytes, neutrophils and eosinophils. On average, eosinophil counts are ∼7× higher among Tsimane individuals compared to current Western clinical standards, presumably reflecting chronic exposure to multicellular parasites, whereas basophil and monocyte counts are lower and might reflect greater recruitment of these cells out of circulation into localized sites of infection [31]. Acute inflammation, measured by CRP, is roughly comparable between US and Tsimane adults aged 18–49, although the etiology differs: among the Tsimane, inflammation is primarily driven by infection, whereas obesity, cigarette smoking and chronic inflammatory disorders (e.g. Type 2 diabetes) are larger contributors to low-grade chronic inflammation in the US population [31].

### Predictions

Considering how the immunological demands of fetal tolerance and pathogen defense may collide in the pathogen-rich, resource-restricted environment currently inhabited by the Tsimane, we suggest three possible scenarios. *Scenario 1*: Tsimane women may experience less pregnancy-induced changes in immune function than do US women, potentially compromising fetal tolerance to maintain pathogen resistance. In this case, we would expect that the estimated effects of pregnancy on leukocyte (i.e. WBC) differential and CRP would be comparatively smaller in magnitude among the Tsimane. *Scenario 2*: Tsimane women may experience similar changes in immunity to women in less pathogenic conditions, thus experiencing greater infection risk at the cost of maintaining fetal tolerance. Under this scenario, both the direction and magnitude of the estimated effects of pregnancy on immune status should be comparable between populations. *Scenario 3*: Tsimane women may compensate for the dual burden of fetal tolerance and infection by preferentially elevating defenses which are less likely to specifically target fetal antigens (e.g. non-specific immunity). In this context, pregnancy should correspond to a disproportionate increase in markers of non-specific immunity (e.g. neutrophils, monocytes and CRP) compared to degree of down-regulation of antigen-specific immune function (e.g. total lymphocyte count).

## METHODOLOGY

### Tsimane health and life history project dataset

Mixed cross-sectional and longitudinal data were collected from 2004 to 2014 as part of the ongoing Tsimane Health and Life History Project (THLHP; http://tsimane.anth.ucsb.edu/index.html, (13 July 2020, date last accessed)) [27, 31–33]. Pregnancy status was determined during medical visits based on date of last menses, with urinary pregnancy tests administered by the physician when pregnancy was suspected. Pregnancies were cross-validated against subsequent annual demographic and census interviews that ascertained children’s birth dates, permitting detection of pregnancies occurring between medical visits and pregnancies that went undetected during previous physician examinations [33].

Venous blood drawn into heparinized vacutainers was analyzed immediately after collection. Total leukocyte counts were determined with a QBC Autoread Plus dry hematology system (QBC Diagnostics) and relative fractions of neutrophils, eosinophils, lymphocytes, basophils and monocytes were determined manually by microscopy with a hemocytometer. Blood drawn into serum vacutainers was allowed to clot and then centrifuged to separate serum, which was frozen in liquid nitrogen and then transported on dry ice for analysis at the University of California, Santa Barbara (UCSB). Serum samples were then analyzed to determine CRP concentration at the UCSB Human Biodemography lab via enzyme immunoassay (ELISA) [34], using a protocol validated against the same University of Washington lab responsible for NHANES data analyses [31].

For this study, the THLHP dataset included pregnant and non-pregnant cycling women aged 18–45 years with total and differential leukocyte count (WBC) and/or CRP concentration. Non-pregnant women who were not regularly menstruating due to either lactational amenorrhea or menopause were excluded from analyses. Given these inclusion criteria, the initial THLHP sample contained 1198 observations (cycling *N* = 942; pregnant *N* = 256). The number of pregnant women sampled was lower than the number of cycling women, and pregnant participants were, on average, younger than cycling women. Based on previously reported Tsimane interbirth intervals and THLHP oversampling of people over age 40 during some rounds of data collection, this initial sampling distribution roughly followed the expected 3:1 ratio of cycling to pregnant participants. To address potential sampling biases, we randomly selected a subset of cycling women from the initial dataset with an age-distribution matching that of the pregnant subset. We did this by dividing the pregnant subset into 6-year age groups and using the* sample* function in R to pick a proportional sample of cycling women from each age bracket. Since not all individuals had recorded data for both CRP and WBC, we conducted this age-matching process separately for participants with complete WBC differential data and those with CRP data. Across the final aggregated dataset used for this study, slightly more individuals were sampled during the second and third trimester compared to the first, although not more than would be expected by chance (*χ*^2^ = 3.66, df = 2, *P* > 0.1). Pregnant and cycling Tsimane women exhibited comparable body mass index (BMI) (*t* = −0.73, df = 497.01, *P*-value = 0.46), while median parity (i.e. number of live births) was higher among pregnant women (*t* = −3.20, df = 454.55, *P*-value <0.01).

Although median parity was comparatively lower within the cycling sample, this was likely due to the mixed cross-sectional and longitudinal nature of the THLHP collection schedule rather than disproportionate sampling of individuals with undiagnosed fertility issues. Based on the ratio of Tsimane women experiencing lactational amenorrhea to those who are currently pregnant (at any given age), lactational amenorrhea usually lasts between 12 and 14 months. Considering the average interbirth interval in this population is ∼30 months [28], these estimates indicate that most Tsimane women experience ∼7 to 9 months of regular cycling between each pregnancy. In our entire sample of non-pregnant cycling Tsimane women, the median number of recorded live births was 4 (range = 0–11), providing further evidence that by-and-large these individuals were simply in-between pregnancies when these particular data were collected. Only 88 out of 727 total observations in the cycling sample belonged to nulliparous individuals, and of these the majority belonged to individuals who were under the age of 25. In total, only ∼3% of non-pregnant regularly cycling Tsimane women in our sample were both nulliparous and over the age 25, suggesting that our findings are not skewed by disproportionate sampling of women with undiagnosed infertility. For complete descriptive statistics, see Table 2.

**Table 2.** Descriptive statistics indicating median value and range (in parentheses) for age, BMI and parity (number of live births), as well as number of repeat samples and total sample size for each population, reproductive status and data subset (leukocyte differential/CRP)

Subset	Population	Descriptive	Cycling	T1	T2	T3
Leukocyte differential	NHANES	Age	27 (20–44)	25 (18–41)	27 (19–40)	28 (18–39)
	THLHP		32 (18–45)	32 (18–45)	29 (18–45)	34 (18–45)
	NHANES	BMI	26 (15–73)	26 (17–67)	27 (19–57)	30 (21–57)
	THLHP		24 (17–36)	23 (17–31)	23 (19–37)	25 (19–33)
	NHANES	Parity	0 (0, 3)	2 (0, 3)	1 (0, 3)	2 (1, 3)
	THLHP		4 (0, 10)	6 (1, 11)	5 (0, 11)	6 (1, 11)
	NHANES	RM	0	0	0	0
	THLHP		149	1	3	6
	NHANES	*N*	1060 (71.41% np)	52	107	108
	THLHP		662 (13.14% np)	70	94	91
CRP	NHANES	Age	27 (20–45)	25 (18–41)	27 (19–43)	28 (18–39)
	THLHP		38 (18–45)	38 (21–44)	37 (22–43)	40 (19–42)
	NHANES	BMI	26 (15–73)	26 (17–67)	27 (19–57)	30 (21–57)
	THLHP		24 (19–37)	22 (21–29)	25 (22–32)	26 (23–30)
	NHANES	Parity	0 (0, 3)	2 (0, 3)	1 (0, 3)	1 (1, 3)
	THLHP		6 (0, 11)	7 (2, 12)	8 (3, 10)	8 (1, 12)
	NHANES	RM	0	0	0	0
	THLHP		0	0	0	0
	NHANES	*N*	476 (69.75% np)	53	113	110
	THLHP		65 (10.77% np)	10	11	8
Total	NHANES	*N*	1536	105	220	218
	THLHP		727	80	105	99

‘% np’ indicates percent of nulliparous individuals within sample.

T1, trimester 1; T2, trimester 2; T3, trimester 3.

### NHANES dataset

We compiled data from NHANES (2003–16) (https://wwwn.cdc.gov/nchs/nhanes/, (14 July 2020, date last accessed)), limiting our sample to pregnant and cycling women between ages 18 and 45 years with complete data on WBC differential and/or CRP concentration. To ensure as comparable of a selection process as possible between THLHP and NHANES samples, individuals in the NHANES dataset were included in the cycling group if they were not pregnant or breastfeeding at the time of the study, reported having regular menstrual cycles throughout the last year, and were not currently using hormonal contraception. No exclusions were based on medical diagnoses. CRP was measured using nephelometry, while total and differential leukocyte counts were measured using the Coulter method. All participants in the NHANES dataset were measured only once. According to these inclusion criteria and dataset characteristics, the initial NHANES dataset selected for this study contained 1652 observations (cycling *N* = 1375; pregnant *N* = 277). The same method used to age-match groups within the THLHP dataset was utilized to age-match cycling and pregnant subsets within the NHANES dataset, for both WBC differential and CRP subsamples. Pregnancy was determined by participant report or urine test at the time of exam, which may explain why women in their first trimester were significantly under-sampled relative to women in later pregnancy (*χ*^2^ = 25.15, df = 2, *P* < 0.001). Overall, pregnant US women exhibited higher BMI (*t* = −2.59, df = 490.17, *P*-value <0.01) and lower median parity (*t* = −13.91, df = 487.45, *P*-value <0.001) than non-pregnant women. For complete descriptive statistics, see Table 2.

Given population-level differences in age (*t* = −13.73, df = 1460.40, *P*-value <0.001), BMI (*t* = 18.41, df = 1957.40, *P*-value <0.001) and parity (*t* = −36.27, df = 1107.40, *P*-value <0.001), we did not attempt to match sampling distributions across populations, as this would have substantially reduced our overall sample for analysis. Instead, we controlled for age, BMI and parity in all statistical models. The final dataset is available at https://doi.org/10.25349/D94C77, (14 July 2020, date last accessed).

### Statistical analyses

All analyses were conducted in R 3.6.2 (https://cran.r-project.org/, (14 July 2020, date last accessed)) using the *brms* package [35]. For each outcome variable, we estimated the population-specific effects of reproductive status (i.e. non-pregnant cycling, trimester 1, trimester 2 or trimester 3), adjusting for fixed-effects of age, BMI and parity and, when applicable, a random effect of participant ID, to account for repeated measures per participant in the mixed cross-sectional and longitudinal THLHP sample. Due to population-specific differences in immune marker distributions, THLHP and NHANES eosinophil count, monocyte count and monocyte proportion were modeled separately using discrete priors and different distribution families. Among the Tsimane, raw eosinophil counts were log-transformed and then estimated using a Gaussian model, while eosinophil counts among US women followed a negative binomial distribution and were modeled as such. Monocyte counts among Tsimane women included excess zeroes and were therefore approximated using a zero-inflated Poisson model, while monocyte counts among US women were log-transformed and estimated using a Gaussian distribution. All other biomarkers were distributed similarly across populations and were therefore modeled together, with *population* as a model term and *trimester:population* as the interaction term. Gaussian models were used to estimate all log-transformed measures following a normal distribution (i.e. CRP, total leukocyte count, neutrophil count and proportion, lymphocyte count and proportion) while zero-inflated Poisson models were used for biomarkers with excess zero counts (i.e. basophil count). In both populations, eosinophil percentage was log-left-skewed and was modeled using a skew-normal distribution following log-transformation.

Median values and 5th and 95th quantiles for all outcome variables in each reproductive state were estimated using age-, parity- and BMI-standardized posterior population distributions (Table 3). Delta values, both between populations for each reproductive state (Table 3) and between reproductive states within each population (Table 4), were calculated as the difference between standardized posterior parameter distributions, with the exception of basophil and monocyte counts and proportions, which were calculated as the difference between standardized posterior population distributions to account for excess zeros. When reporting results, posterior distributions on log scales were exponentiated to return them to the linear scale of the original measures. Estimated fixed-effects of age, parity and BMI for each outcome variable are provided in Table 5.

**Table 3.** Leukocyte counts, leukocyte proportions and CRP concentration by population and reproductive status

Measure	Population	Cycling	Trimester 1	Trimester 2	Trimester 3
Total leukocytes (cells/µl)	NHANES	7013 (4473, 10 885)	8154 (5172, 12 769)	9132 (5833, 14 450)	9056 (5825, 14 338)
	THLHP	9392 (6112, 14 788)	9733 (6311, 15 432)	9248 (6015, 14 644)	9109 (5869, 14 353)
	Δ	2439 (2182, 2690)	1661 (894, 2408)	135 (−441, 755)	44 (−563, 627)
		34.8% (30.8%, 38.6%)	20.5% (10.6%, 31.1%)	1.5% (−4.7%, 8.4%)	0.5% (−6.0%, 7.1%)
Neutrophils (cells/µl)	NHANES	3914 (2156, 7080)	5158 (2886, 9356)	6602 (3736, 11 741)	6439 (3593, 11 821)
	THLHP	4866 (2728, 8556)	5265 (2946, 9432)	5762 (3191, 10 029)	5855 (3248, 10 507)
	Δ	872 (704, 1049)	83 (−488, 673)	−758 (−1289, −260)	−652 (−1170, −107)
		22.0% (17.5%, 26.8%)	1.6% (−8.8%, 14.0%)	−11.6% (−19.0%, −4.1%)	−10.1% (−17.4%, −1.7%)
Lymphocytes (cells/µl)	NHANES	2178 (1313, 3606)	1980 (1184, 3282)	1836 (1080, 3092)	1704 (1020, 2900)
	THLHP	2768 (1678, 4603)	2694 (1599, 4501)	2159 (1290, 3600)	2169 (1296, 3727)
	Δ	568 (479, 659)	715 (489, 935)	324 (165, 486)	480 (329, 637)
		26.1% (21.8%, 30.5%)	36.5% (23.7%, 49.8%)	17.7% (8.6%, 27.3%)	28.5% (18.9%, 38.8%)
Eosinophils (cells/µl)	NHANES	125 (10, 520)	133 (11, 535)	106 (9, 447)	104 (10, 419)
	THLHP	1176 (249, 5669)	1315 (281, 5909)	947 (196, 4268)	695 (152, 3200)
	Δ	1016 (914, 1139)	1095 (856, 1404)	784 (629, 973)	553 (432, 703)
		555.1% (490.9%, 630.7%)	572.9% (400.7%, 826.9%)	514.3% (388.5%, 680.4%)	379.3% (272.3%, 515.4%)
Monocytes (cells/µl)	NHANES	493 (287, 837)	537 (323, 918)	550 (319, 936)	609 (355, 1020)
	THLHP	0 (0, 136)	0 (0, 168)	0 (0, 92)	0 (0, 126)
	Δ	−457 (−814, −224)	−494 (−892, −235)	−516 (−900, −287)	−577 (−981, −299)
Basophils (cells/µl)	NHANES	0 (0, 113)	0 (0, 114)	0 (0, 118)	0 (0, 112)
	THLHP	0 (0, 0)	0 (0, 0)	0 (0, 0)	0 (0, 0)
	Δ	0 (−113, 0)	0 (−114, 0)	0 (−114, 0)	0 (−112, 0)
% Neutrophil	NHANES	56.4% (41.9%, 76.7%)	63.9% (47.6%, 84.9%)	71.0% (52.7%, 93.9%)	71.8% (53.6%, 95.6%)
	THLHP	51.1% (38.0%, 68.9%)	53.9% (40.4%, 72.4%)	61.8% (45.7%, 82.6%)	64.4% (47.7%, 86.5%)
	Δ	−5.2% (−6.3%, −4.1%)	−9.8% (−13.3%, −6.7%)	−9.2% (−12.1%, −6.4%)	−7.3% (−10.3%, −4.2%)
% Lymphocytes	NHANES	30.7% (20.1%, 47.4%)	24.4% (15.9%, 36.5%)	19.6% (12.7%, 30.8%)	18.6% (12.3%, 29.1%)
	THLHP	28.8% (19.2%, 43.9%)	27.3% (17.5%, 41.8%)	23.3% (15.0%, 35.8%)	23.6% (15.2%, 36.8%)
	Δ	−2.0% (−2.9%, −1.1%)	3.1% (0.9%, 5.3%)	3.2% (1.8%, 4.7%)	5.2% (3.8%, 6.7%)
% Eosinophils	NHANES	3.2% (1.2%, 7.1%)	2.8% (1.1%, 6.2%)	2.4% (0.9%, 5.4%)	2.3% (0.9%, 5.4%)
	THLHP	15.1% (5.7%, 33.3%)	14.5% (5.4%, 32.7%)	11.8% (4.4%, 26.5%)	9.5% (3.5%, 20.8%)
	Δ	11.5% (10.8%, 12.2%)	11.2% (9.7%, 12.8%)	9.0% (8.0%, 10.2%)	6.8% (5.9%, 7.7%)
% Monocytes	NHANES	7.5% (4.9%, 11.6%)	7.2% (4.6%, 11.2%)	6.4% (4.1%, 9.9%)	7.2% (4.7%, 11.2%)
	THLHP	0.0% (0.0%, 2.0%)	0.0% (0.0%, 2.0%)	0.0% (0.0%, 2.0%)	0.0% (0.0%, 2.0%)
	Δ	−7.2% (−11.3%, −4.2%)	−6.9% (−10.9%, −3.9%)	−6.9% (−11.0%, −4.1%)	−6.9% (−11.1%, −4.0%)
% Basophils	NHANES	0.0% (0.0%, 2.0%)	0.0% (0.0%, 1.0%)	0.0% (0.0%, 1.0%)	0.0% (0.0%, 1.0%)
	THLHP	0.0% (0.0%, 0.0%)	0.0% (0.0%, 0.0%)	0.0% (0.0%, 0.0%)	0.0% (0.0%, 0.0%)
	Δ	0.0% (−2.0%, 0.0%)	0.0% (−1.0%, 0.0%)	0.0% (−1.0%, 0.0%)	0.0% (−1.0%, 0.0%)
CRP (mg/l)	NHANES	1.48 (0.25, 8.08)	4.03 (0.70, 23.57)	5.04 (0.87, 30.26)	3.47 (0.59, 21.19)
	THLHP	1.32 (0.24, 7.64)	3.92 (0.60, 24.56)	3.91 (0.65, 24.68)	1.67 (0.24, 9.68)
	Δ	−0.13 (−0.51, 0.40)	−0.11 (−2.30, 3.34)	−1.04 (−3.00, 2.25)	−1.89 (−2.85, −0.25)
		−8.4% (−33.1%, 27.4%)	−2.8% (−52.7%, 91.7%)	−20.2% (−56.0%, 46.2%)	−54.1% (−75.8%, −6.7%)

For all measures, reported absolute values are the estimated median, 5th and 95th percentiles of the posterior *population* distributions, standardized by age, BMI and parity. Estimated median, 5th and 95th percentiles for delta values (Δ) represent the calculated difference between standardized *parameter* distributions, with the exception of monocytes and basophils, which were calculated using the difference between posterior *population* distributions to include excess zero values (and therefore produce larger confidence intervals).

**Table 4.** Model-estimated delta values (Δ) for leukocyte counts and CRP, by trimester and population

Measure	Population	Unit	T1–cycling Δ	T2–cycling Δ	T3–cycling Δ
Total leukocytes	NHANES	cells/µl	1058 (552, 1591)	2183 (1756, 2594)	2048 (1641, 2448)
		%	15.1% (7.8%, 22.8%)	31.1% (24.9%, 37.1%)	29.2% (23.2%, 34.9%)
	THLHP	cells/µl	285 (−277, 838)	−121 (−596, 364)	−350 (−805, 118)
		%	3.0% (−2.9%, 8.9%)	−1.3% (−6.3%, 3.9%)	−3.7% (−8.5%, 1.3%)
Neutrophils	NHANES	cells/µl	1203 (781, 1637)	2580 (2210, 2941)	2500 (2141, 2886)
		%	30.3% (19.5%, 41.3%)	65.0% (55.5%, 74.4%)	63.0% (53.7%, 72.9%)
	THLHP	cells/µl	410 (13, 815)	934 (561, 1319)	989 (613, 1372)
		%	8.4% (0.3%, 16.7%)	19.3% (11.6%, 27.5%)	20.5% (12.5%, 28.4%)
Lymphocytes	NHANES	cells/µl	−219 (−348, −78)	−343 (−441, −238)	−497 (−585, −402)
		%	−10.0% (−15.9%, −3.6%)	−15.7% (−20.1%, −11.0%)	−22.8% (−26.7%, −18.6%)
	THLHP	cells/µl	−73 (−254, 101)	−584 (−717, −455)	−580 (−705, −448)
		%	−2.7% (−9.2%, 3.7%)	−21.3% (−25.9%, −16.7%)	−21.2% (−25.5%, −16.5%)
Eosinophils	NHANES	cells/µl	8 (−31, 55)	−31 (−55, −4)	−37 (−60, −9)
		%	4.2% (−17.0%, 30.3%)	−17.0% (−29.5%, −2.1%)	−19.8% (−32.4%, −5.0%)
	THLHP	cells/µl	87 (−142, 364)	−264 (−421, −93)	−499 (−635, −353)
		%	7.2% (−11.9%, 30.1%)	−22.0% (−34.3%, −7.7%)	−41.7% (−51.1%, −30.3%)
Monocytes	NHANES	cells/µl	45 (4, 85)	49 (20, 81)	105 (72, 138)
		%	9.0% (0.9%, 17.3%)	10.0% (4.0%, 16.5%)	21.2% (14.6%, 28.1%)
	THLHP	cells/µl	0 (−132, 164)	0 (−133, 89)	0 (−133, 122)
Basophils	NHANES	cells/µl	0 (−110, 110)	0 (−110, 115)	0 (−110, 108)
	THLHP	cells/µl	0 (0, 0)	0 (0, 0)	0 (0, 0)
CRP	NHANES	mg/l	2.49 (1.61, 3.54)	3.66 (2.81, 4.62)	2.04 (1.50, 2.66)
		%	169.0% (105.9%, 244.7%)	247.2% (184.6%, 318.0%)	137.7% (98.0%, 184.2%)
	THLHP	mg/l	2.45 (0.67, 5.85)	2.73 (0.95, 5.84)	0.26 (−0.58, 1.80)
		%	180.7% (46.1%, 436.6%)	199.3% (65.1%, 447.3%)	19.1% (−38.2%, 138.8%)

For each trimester, delta was calculated as the difference from non-pregnant cycling. Estimated median, 5th and 95th percentiles for delta values were estimated as the difference between parameter distributions, with the exception of THLHP monocytes and NHANES and THLHP basophils, which were calculated using the difference between posterior population distributions to include excess zero values.

**Table 5.** Median and 5% and 95% confidence intervals (in parentheses) for the estimated fixed-effects of age, BMI and parity for all models

Measure	Units	Age	BMI	Parity
Total leukocytes	ln (cells/µl)	−0.001 (−0.003, 0.001)	0.009 (0.007, 0.010)	−0.004 (−0.009, 0.001)
Neutrophils	ln (cells/µl)	−0.001 (−0.003, 0.001)	0.011 (0.009, 0.013)	−0.009 (−0.016, −0.002)
Lymphocytes	ln (cells/µl)	−0.003 (−0.005, −0.001)	0.007 (0.006, 0.009)	0.000 (−0.006, 0.006)
Eosinophils (THLHP)	ln (cells/µl)	0.014 (0.004, 0.024)	−0.006 (−0.023, 0.011)	−0.003 (−0.028, 0.021)
Eosinophils (NHANES)	cells/µl	−0.008 (−0.017, 0.001)	0.005 (−0.000, 0.011)	0.009 (−0.028, 0.047)
Monocytes (THLHP)	cells/µl	−0.022 (−0.024, −0.020)	0.023 (0.019, 0.026)	0.056 (0.051, 0.061)
Monocytes (NHANES)	ln (cells/µl)	−0.001 (−0.004, 0.002)	0.003 (0.001, 0.005)	−0.033 (−0.047, −0.021)
Basophils	cells/µl	0.007 (0.006, 0.009)	0.013 (0.013, 0.014)	−0.036 (−0.041, −0.030)
% Neutrophils	ln (% cells/µl)	0.000 (−0.001, 0.001)	0.002 (0.001, 0.003)	−0.005 (−0.009, −0.002)
% Lymphocytes	ln (% cells/µl)	−0.002 (−0.004, −0.000)	−0.002 (−0.003, −0.000)	0.004 (−0.002, 0.009)
% Eosinophils	ln (% cells/µl)	0.001 (−0.002, 0.005)	−0.003 (−0.005, 0.000)	0.017 (0.007, 0.027)
% Monocytes (NHANES)	ln (% cells/µl)	−0.001 (−0.004, 0.001)	−0.006 (−0.007, −0.004)	−0.021 (−0.031, −0.011)
% Monocytes (THLHP)	% cells/µl	0.020 (0.002, 0.038)	0.017 (−0.012, 0.045)	−0.002 (−0.044, 0.042)
% Basophils	% cells/µl	0.011 (−0.004, 0.025)	0.001 (−0.008, 0.010)	0.004 (−0.057, 0.061)
CRP	ln (mg/l)	−0.000 (−0.012, 0.011)	0.085 (0.077, 0.092)	0.036 (−0.006, 0.078)

## RESULTS

### Total leukocyte counts overlap among second and third trimester Tsimane and US women, despite Tsimane women having substantially elevated non-pregnant baselines

**Figure 1.**
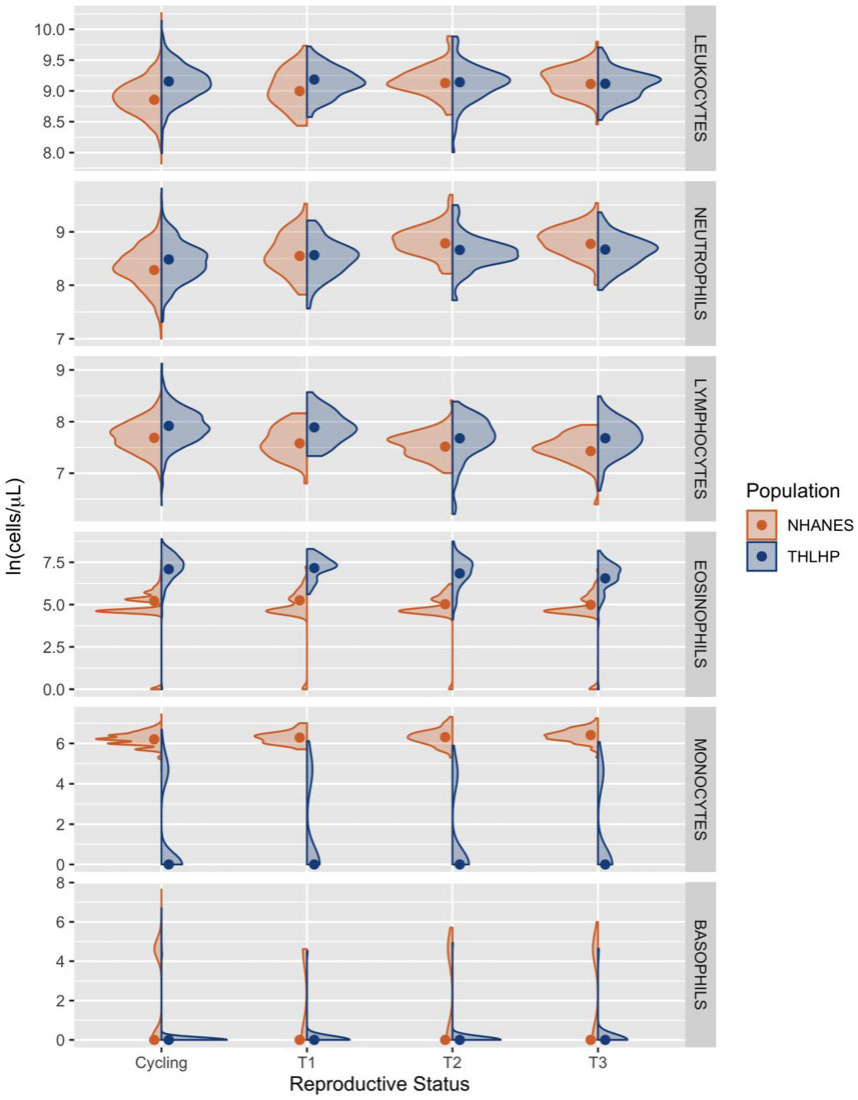
Total leukocyte, neutrophil, lymphocyte, eosinophil, monocyte and basophil counts among Tsimane (blue) and US women (orange) in each reproductive state. Raw data are represented by density curves, while dots indicate model-estimated median values standardized by age, BMI and parity. C, non-pregnant cycling; T1, trimester 1; T2, trimester 2; T3, trimester 3. All *y*-axes are natural logged but are on different scales

Non-pregnant cycling Tsimane women had, on average, 34.8% (CI = 30.8%, 38.6%) or 2439 cells/µl (CI = 2182, 2690) more circulating leukocytes than their US peers—reflecting elevated baseline immune activation (Table 3). Among US women, first, second and third trimester leukocyte counts were increased over non-pregnant baseline by 15.1% (CI = 7.8%, 22.8%), 31.1% (CI = 24.9%, 37.1%) and 29.2% (CI = 23.2%, 34.9%), respectively (Table 4). In contrast, there was no appreciable variation in leukocyte count among Tsimane women attributable to reproductive state. As a result, leukocyte counts did not differ significantly between Tsimane and US women in the second and third trimester (Table 3 and Fig. 1).

### Pregnant women in both populations exhibit elevated neutrophil counts, reduced lymphocytes and fewer eosinophils than non-pregnant women, but the magnitude of these differences varies by trimester and population

#### Neutrophils

Neutrophil counts among cycling Tsimane women were 22.0% (CI = 17.5%, 26.8%) or 872 cells/µl (CI = 704, 1049) higher than cycling US women (Table 3). Within the US sample, women in the first, second and third trimester possessed neutrophil counts that were elevated over non-pregnant baseline by 30.3% (CI = 19.5%, 41.3%), 65.0% (CI = 55.5%, 74.4%) and 63.0% (CI = 53.7%, 72.9%), respectively (Table 4). Pregnancy was also associated with elevated neutrophil counts among Tsimane women, although the magnitude of this relationship was relatively attenuated: neutrophil counts were increased by 8.4% (CI = 0.3%, 16.7%), 19.3% (CI = 11.6%, 27.5%) and 20.5% (CI = 12.5%, 28.4%) among first, second and third trimester Tsimane women (Table 4). Consequently, average neutrophil counts among second and third trimester Tsimane women were 758 (CI = 260, 1289) and 652 (CI = 107, 1170) cells/µl lower than those exhibited by US women (Table 3 and Fig. 1).

#### Lymphocytes

Lymphocyte counts among non-pregnant Tsimane women were 26.1% (CI = 21.8%, 30.5%) or 568 cells/µl (CI = 479, 659) greater than those observed among cycling US women (Table 3). Within the US sample, first, second and third trimester lymphocyte counts were reduced by 10.0% (CI = 3.6%, 15.9%), 15.7% (CI = 11.0%, 20.1%) and 22.8% (CI = 18.6%, 26.7%) compared to non-pregnant baselines (Table 4). Among Tsimane women, there was also a negative relationship between trimester and lymphocyte count: second and third trimester lymphocyte counts were reduced by 21.3% (CI = 16.7%, 25.9%) and 21.2% (CI = 16.5%, 25.5%), respectively (Table 4 and Fig. 1).

#### Eosinophils

Eosinophil counts among cycling Tsimane women were 555.1% (CI = 490.9%, 630.7%) or 1016 cells/µl (CI = 914, 1139) higher than cycling women in the USA (Table 3). Among US women, there was a negative relationship between eosinophil count and pregnancy progression, with counts unchanged in the first trimester, but reduced by 17.0% (CI = 2.1%, 29.5%) in the second trimester and 19.8% (CI = 5.0%, 32.4%) in the third trimester (Table 4). Among Tsimane women, first trimester eosinophil counts did not significantly differ from cycling, while second and third trimester counts were 22.0% (CI = 7.7%, 34.3%) and 41.7% (CI = 30.3%, 51.1%) lower than non-pregnant baseline, respectively (Table 4 and Fig. 1). Regardless of these greater percent reductions from cycling, third trimester eosinophils remained 379.3% (CI = 272.3%, 515.4%) higher in Tsimane women than US women.

#### Monocytes

Among Tsimane women, monocyte counts did not vary by reproductive state and remained consistently lower than those among US women (Table 3). Within the US sample, monocyte counts in the first, second and third trimester counts were increasingly elevated above cycling baseline by 9.0% (CI = 0.9%, 17.3%), 10.0% (CI = 4.0%, 16.5%) and 21.2% (CI = 14.6%, 28.1%) (Table 4 and Fig. 1).

#### Basophils

In both populations, basophil count remained low and did not vary by reproductive state (Tables 3 and 4; Fig. 1).

### Population-specific differences in immune cell counts produce variation in leukocyte proportions

**Figure 2.**
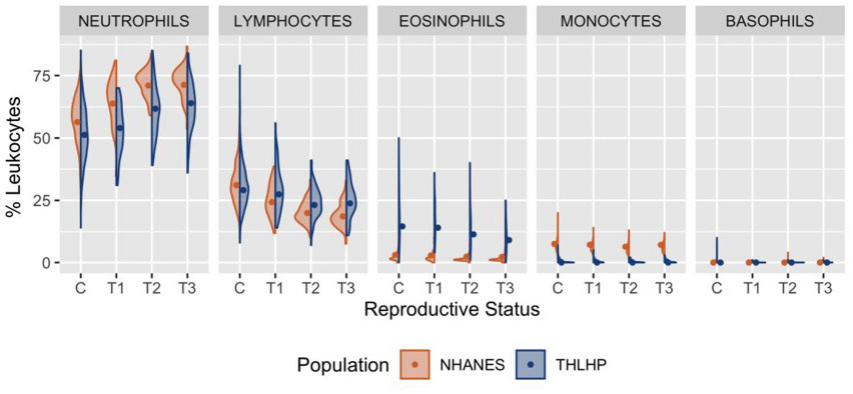
Leukocyte proportions among Tsimane (blue) and US women (orange) across reproductive states. Raw data are represented by density curves, while dots indicate model-estimated median values standardized by age, BMI and parity. C, non-pregnant cycling; T1, trimester 1; T2, trimester 2; T3, trimester 3

Among cycling Tsimane women, total leukocyte count comprised 51.1% neutrophils (CI = 38.0%, 68.9%), 28.8% lymphocytes (CI = 19.2%, 43.9%), 15.1% eosinophils (CI = 5.7%, 33.3%) and <1% monocytes and basophils (Table 3). In comparison, leukocyte counts among cycling US women comprised 56.4% neutrophils (CI = 41.9%, 76.7%), 30.7% lymphocytes (CI = 20.1%, 47.4%), 7.5% monocytes (CI = 4.9%, 11.6%), 3.2% eosinophils (CI = 1.2%, 7.1%) and <1% basophils (Table 3 and Fig. 2).

#### Neutrophils

In both populations neutrophil proportions were increasingly elevated by trimester, peaking at 71.8% (CI = 53.6%, 95.6%) among US women and 64.4% (CI = 47.7%, 86.5%) among Tsimane women (Table 3 and Fig. 2). Across all reproductive states, US women displayed greater neutrophil proportions than Tsimane women (Table 3).

#### Lymphocytes

Pregnancy progression was associated with declining lymphocyte proportions among both Tsimane and US women, though Tsimane individuals exhibited higher lymphocyte proportions across all three trimesters. Lymphocyte percentages reached population-specific lows of 23.3% (CI = 15.0%, 35.8%) for Tsimane women in the second trimester and 18.6% (CI = 12.3%, 29.1%) for US women in the third trimester (Table 3 and Fig. 2).

#### Eosinophils

Later pregnancy was associated with reduced eosinophil proportions among the Tsimane, reaching a third-trimester low of 9.5% (CI = 3.5%, 20.8%). Among US women, pregnancy progression was associated with a comparatively small decrease in eosinophil proportion, reaching a third-trimester low of 2.3% (CI = 0.9%, 5.4%) (Table 3 and Fig. 2).

#### Monocytes and basophils

In both populations, basophil and monocyte proportions were low and did not vary substantially by reproductive state (Table 3 and Fig. 2).

### Early pregnancy is associated with inflammatory activation in both populations, but only US women retain elevated inflammation in late pregnancy

**Figure 3.**
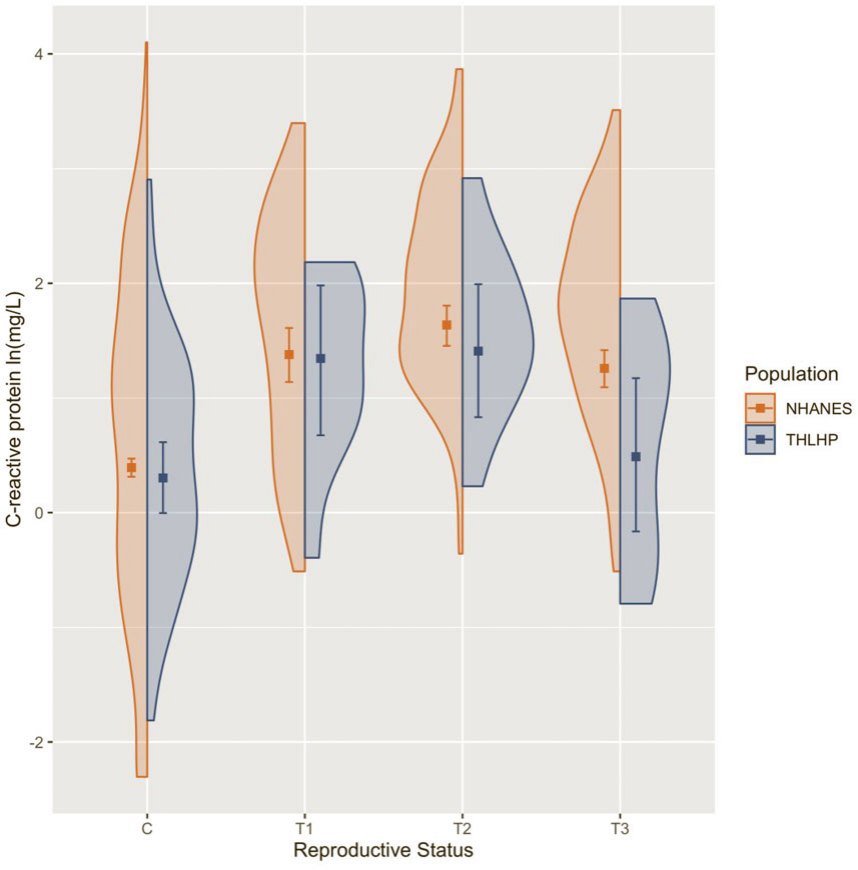
CRP concentration among Tsimane (blue) and US women (orange) by reproductive status. Raw data are represented by density curves. Dots indicate model-estimated median values and error bars represent model-estimated 5th and 95th confidence intervals, all standardized for age, BMI and parity. C, non-pregnant cycling; T1, trimester 1; T2, trimester 2; T3, trimester 3. *y*-Axis is natural logged

Among cycling women, CRP concentration did not differ substantially across populations (Table 3). Among US women in the first, second and third trimester, CRP was elevated by 169.0% (CI = 105.9%, 244.7%), 247.2% (CI = 184.6%, 318.0%) and 137.7% (CI = 98.0%, 184.2%), respectively (Table 4). Among Tsimane women, CRP in the first and second trimester was elevated by 180.7% (CI = 46.1%, 436.6%) and 199.3% (CI = 65.1%, 447.3%), while third trimester CRP largely overlapped with baseline concentrations (Table 4). Consequently, third trimester Tsimane women displayed CRP concentrations that were, on average, 54.1% (CI = 6.7%, 75.8%) or 1.89 mg/l (CI = 0.25, 2.85) lower than their third trimester US counterparts (Table 3 and Fig. 3).

### Reduced models excluding BMI and parity predict even greater magnitude of population-level differences in total leukocyte and neutrophil count and CRP concentration

To further evaluate the contribution of BMI and parity to between-population differences in immune markers across reproductive states, we compared the predicted values for total leukocyte, neutrophil, lymphocyte, eosinophil and monocyte count and CRP concentration generated by our full models (which included age, parity and BMI and *trimester:population* as interaction term) with those produced by reduced models, which omitted BMI and parity as covariates. Models excluding BMI and parity predicted *higher* values for total leukocytes, neutrophils and CRP among US women and *lower* values for total leukocyte and neutrophil counts among Tsimane individuals, resulting in heightened estimated population-level differences across reproductive states for these measures. Removing effects of BMI and parity produced largely unchanged predicted values for lymphocyte, eosinophil and monocyte counts (Supplementary Table S1 and Fig. S1). Taken together, these results indicate that the ‘true’ differences in total leukocyte and neutrophil count and CRP between US and Tsimane women are partially due to BMI and parity but cannot be fully explained by these factors.

## DISCUSSION

These results provide strong evidence that the immunology of pregnancy, a unique physiological state involving exposure to fetal antigens, consistently follows overarching patterns of modulation across diverse human populations but is also influenced by local ecological conditions. Elevated leukocyte count, currently considered a reliable clinical marker of pregnancy progression [17,36], was replicated in our US sample but was not observed among Tsimane women, who exhibited high leukocyte counts regardless of reproductive phase. Pregnancy was strongly associated with increased neutrophil counts, reduced lymphocytes and fewer eosinophils in both populations, suggesting heavier reliance on non-specific immune defenses and down-regulation of antigen-specific immunity across diverse ecologies. However, although population-specific effects of pregnancy on leukocyte subsets were in the same direction, Tsimane women retained greater absolute and relative lymphocyte and eosinophil counts and showed lower absolute and relative neutrophil and monocyte prevalence, irrespective of trimester. Acute systemic inflammation, measured by CRP, was elevated among pregnant women in the first two trimesters in both populations, which is consistent with previous reports suggesting that pregnancy is a controlled state of inflammation [17, 37]. Yet our results also indicate that, when compared to US women, Tsimane women undergo relatively less change in inflammatory activation across gestation, particularly in the third trimester.

Absence of pregnancy-induced leukocytosis, comparatively low neutrophil and monocyte counts, and relatively attenuated acute inflammation among pregnant Tsimane women may be due in part to strong constraints imposed by infection risk and competing demands on energy distribution. Among the Tsimane, ∼10% of all fetal deaths are attributed to maternal infection [38]. While there are surprisingly few data on the rate of infection-induced miscarriages in the USA, many infectious diseases with the greatest impact on miscarriage risk (e.g. malaria, Dengue fever) are endemic to the Amazonian region and relatively rare in high-income post-industrial contexts, suggesting maternal infection poses a proportionally greater threat to pregnancy among the Tsimane [39, 40]. In addition, infection with hookworm (an intestinal parasite endemic to the Amazon) is associated with older age of first birth and reduced lifetime fertility among Tsimane women [33], possibly as a result of conflicting immunological demands during pregnancy [7]. Maintaining elevated immune activation and sustaining pregnancy are metabolically costly processes [29, 41], especially in resource-restricted environments where infection risk is high [42]. Under such constraints, Tsimane women may retain relatively greater investment in lymphocyte and eosinophil cell populations during pregnancy as a strategy to contend with ongoing exposure to quickly evolving pathogens and parasitic invaders [31], necessitating comparatively tempered neutrophil and monocyte proliferation and systemic inflammation despite potential costs (e.g. bacterial infections).

Another interpretation of our findings is that Tsimane women may be, in certain aspects, better primed for pregnancy than their US counterparts. While the Tsimane contend with high infectious disease morbidity and mortality, pathogen exposure also provides benefits, namely finer immunological calibration that safeguards against autoreactivity and hypersensitivity [8]. It stands to reason that this protective effect extends to encounters with fetal antigens during pregnancy, thus dampening responsivity—especially in terms of inflammatory processes. Chronic infection with parasites that induce similar states of immunological tolerance may especially attune the maternal immune system for pregnancy [43]. Infection with roundworm (*Ascaris lumbricoides*), a low-virulence intestinal parasite that stifles host inflammation, has already been linked to earlier age of first reproduction and higher lifetime fertility among Tsimane women [33], providing preliminary support for this perspective. In contrast, exposure to fetal antigens may prompt exaggerated and potentially deleterious inflammatory activation among women in the USA, where opportunities for immune calibration are comparatively scarce. This interpretation is bolstered by our observation that acute systemic inflammation was higher among US women in later pregnancy, a phase of gestation marked by increasing exposure to cell-free fetal DNA [13]. Comparatively elevated investment in neutrophils among pregnant US women could also indicate a greater skew toward inflammatory activation, considering neutrophils often initiate and amplify inflammatory cascades [ 44]. However, pregnancy-induced neutrophil expansion could also be a compensatory mechanism tempering responsivity to fetal antigens, considering neutrophils are also capable of inducing regulatory T cell phenotypes during pregnancy [45].

### Limitations and future directions

While our findings provide clear evidence that ecological variation impacts both non-pregnant immune profiles and immunological investment strategies during pregnancy, the underlying causal pathways and consequences remain points of speculation. Future research should employ longitudinal study designs to definitively link variation in the expression of fetal tolerance with maternal and infant outcomes, as this approach would allow for a better understanding of how much variation stems from differences in immune calibration and how much can be attributed to localized constraints and trade-offs. Given that there are documented individual and population-level differences in placental morphology [46], with more invasive placentation associated with higher levels of cell-free fetal DNA [47], it is theoretically possible that our findings could be related to population-level differences in fetal antigen exposure stemming from differences in fetal size and/or placental phenotype. To further explore this possibility, future studies would benefit from a closer consideration of how levels of cell-free fetal DNA and placental phenotype might affect maternal immune profiles, particularly across ecologically distinct populations. Along a similar vein, we were not able to account for potential population-level differences in hematological parameters (e.g. blood viscosity, plasma volume), which have been found to vary across other ecological clines (e.g. high versus low altitude) [48]. Considering increased blood viscosity during pregnancy could place functional limits on the number of circulating immune cells, documenting and accounting for hematological profile would be a useful addition to future studies. Subsequent research would also benefit from evaluating the effects of pregnancy and environment on a wider breadth of immune biomarkers. Due to dataset restrictions, we were limited to quantifying large-scale differences in immune cell populations and a singular measure of acute inflammation (with a small THLHP sample size), excluding other relevant and more fine-grained indices of immune function (e.g. cytokine profiles, proportion of regulatory T cells, neutrophil oxidative bursts). Lastly, while the ecological landscape currently inhabited by Tsimane women presumably more closely resembles the environmental conditions that prevailed before the agricultural transition and industrialization, the Tsimane do not serve as a universal representation of other past or contemporary non-industrialized populations. Rather, this study highlights the need for future studies to consider variation both across and within non-industrialized contexts, especially since myriad factors combine to produce mosaic ecologies. The USA itself encompasses underappreciated and understudied disparity in environmental circumstances; numerous so-called ‘Third World’ diseases remain endemic in certain rural areas [49] and disproportionately affect marginalized communities within urban centers [50]. Future research on populations where infectious disease burden remains elevated but where the immunoregulatory benefits are obscured by processed diets, physical inactivity and other adverse consequences of ‘modernization’ may yield particularly fruitful insight into how interactions between environmental factors influence maternal immune profiles across reproductive states.

## CONCLUSIONS AND IMPLICATIONS

To our knowledge, this is the first study to estimate the effects of pregnancy on female immune status in a non-industrialized context and the first to directly compare expressions of fetal tolerance between two ecologically distinct populations. Our results replicate previous findings that pregnancy is not a state of immunosuppression, but rather a period of dynamic immune modulation generally favoring non-specific defenses and down-regulated antigen-specific immunity and parasite response. While these overarching patterns appear to be shared across populations, this study provides evidence that immunological profiles during pregnancy vary in response to ecology, with pregnant Tsimane women exhibiting comparatively elevated investment in lymphocyte and eosinophil cell populations, relatively smaller neutrophil and monocyte counts, and attenuated acute inflammatory activation. These findings likely reflect steep trade-offs between fetal tolerance, pathogen defense and energy restriction among the Tsimane, but could also indicate that Tsimane women benefit from comparatively reduced inflammatory activation and stronger resistance to intracellular pathogens across gestation. Future research is needed to parse out the potential benefits of pathogen exposure in moderating responses to fetal antigens and the constraints imposed by ongoing disease risk and resource budgeting. While such questions remain, this study is an important first step in acknowledging that the immunology of successful pregnancy is not a one-size-fits-all phenomenon. In sum, our findings add nuance to the current understanding of ‘normal’ pregnancy and will hopefully prompt further consideration of ecological variability both across and within populations, especially as it pertains to maternal health and disease.

## Supplementary data

Supplementary data are available at *EMPH* online.

## Supplementary Material

eoaa022_Supplementary_Data
