# Supplementary material for: Immune function during pregnancy varies between ecologically distinct populations
Source: Evol Med Public Health. 2020 Jul 3;2020(1):114–28. doi: 10.1093/emph/eoaa022 (PMC7502269; doi:10.1093/emph/eoaa022)
Supplement: eoaa022_Supplementary_Data [file eoaa022_supplementary_data.pdf]

Figure S1: Natural logged posterior population distributions generated by full and reduced models for total leukocyte (WBC), neutrophil (NEU), lymphocyte (LYM), eosinophil (EOS), and monocyte (MON) count and CRP concentration among Tsimane (blue) and US women (orange). Dots represent posterior population estimated median values. Full models included the fixed-effects of age, BMI, and parity and the random-effects of PID, while reduced models omitted both BMI and parity. C = non-pregnant cycling; T1 = Trimester 1; T2 = Trimester 2; T3 = Trimester 3.

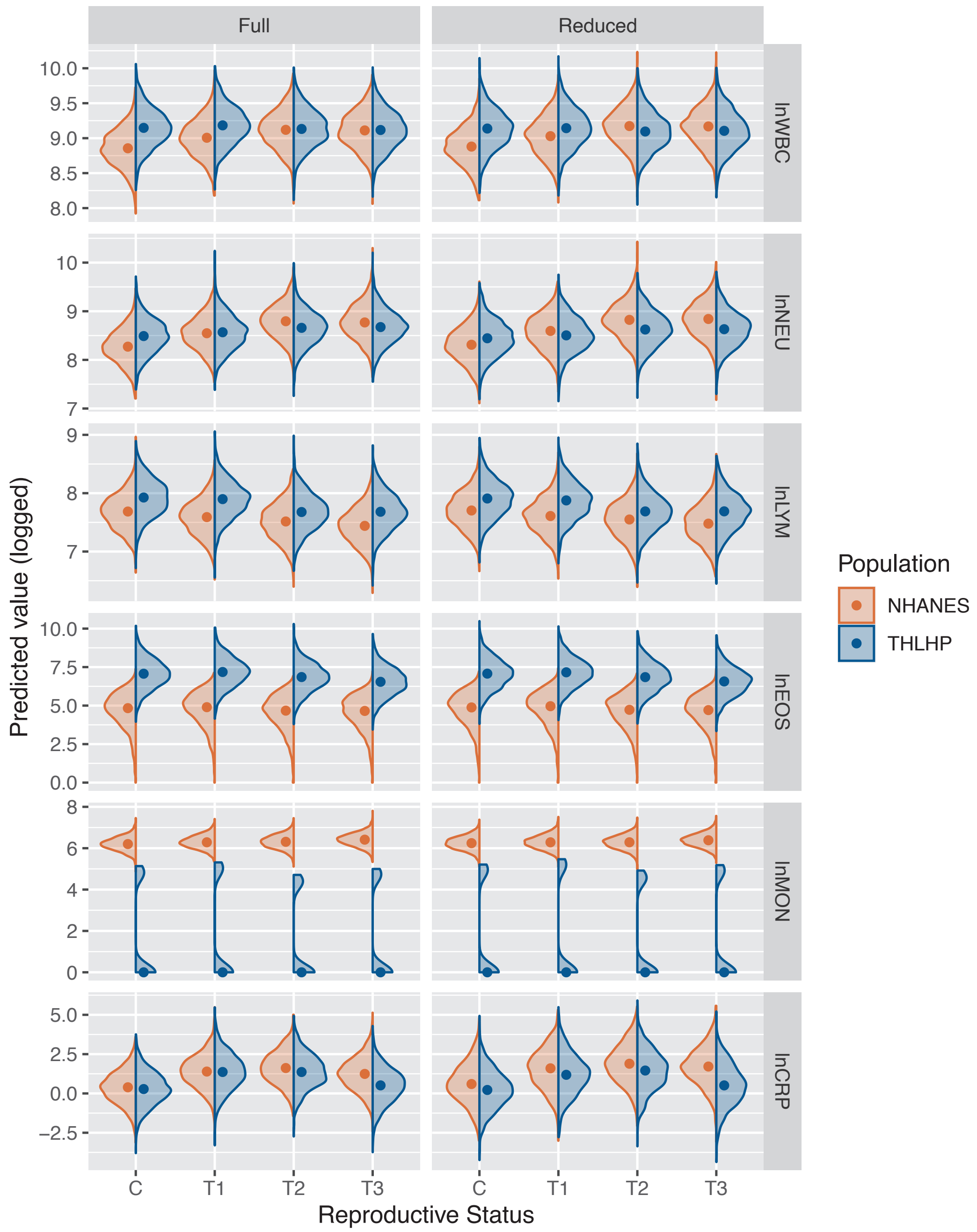

Table S1. Predicted median, 5% and 98% confidence intervals for each measure by population and reproductive status, derived from full and reduced models. Full models included age, random effects of PID, BMI, and parity; reduced models omitted BMI and parity.

Measure	Population	Model	Cycling	Trimester 1	Trimester 2	Trimester 3
WBC	NHANES	Full	7013 (4473,10885)	8154 (5172,12769)	9132 (5833,14450)	9056 (5825,14338)
	THLHP		9392 (6112,14788)	9733 (6311,15432)	9248 (6015,14644)	9109 (5869,14353)
	NHANES	Reduced	7186 (4568,11417)	8341 (5175,13154)	9636 (6041,15002)	9585 (6006,15143)
	THLHP		9300 (5834,14605)	9354 (6017,14908)	8914 (5782,14373)	9002 (5752,14310)
NEU	NHANES	Full	3914 (2156,7080)	5158 (2886,9356)	6602 (3736,11741)	6439 (3593,11821)
	THLHP		4866 (2728,8556)	5265 (2946,9432)	5762 (3191,10029)	5855 (3248,10507)
	NHANES	Reduced	4070 (2187,7382)	5409 (2908,9547)	6789 (3678,12658)	6916 (3733,12464)
	THLHP		4650 (2604,8524)	4937 (2727,9032)	5569 (3074,10169)	5608 (3096,10303)
LYM	NHANES	Full	2178 (1313,3606)	1980 (1184,3282)	1836 (1080,3092)	1704 (1020,2900)
	THLHP		2768 (1678,4603)	2694 (1599,4501)	2159 (1290,3600)	2169 (1296,3727)
	NHANES	Reduced	2217 (1335,3757)	2016 (1212,3375)	1900 (1087,3199)	1770 (1027,3021)
	THLHP		2723 (1625,4591)	2634 (1600,4437)	2183 (1305,3710)	2184 (1282,3715)
EOS	NHANES	Full	125 (10,520)	133 (11,535)	106 (9,447)	104 (10,419)
	THLHP		1176 (249,5669)	1315 (281,5909)	947 (196,4268)	695 (152,3200)
	NHANES	Reduced	131 (11,529)	142 (12,561)	112 (11,489)	110 (9,450)
	THLHP		1182 (244,5596)	1291 (268,5968)	943 (195,4398)	714 (155,3297)
MON	NHANES	Full	493 (287,837)	537 (323,918)	550 (319,936)	609 (355,1020)
	THLHP		0 (0,136)	0 (0,168)	0 (0,92)	0 (0,126)
	NHANES	Reduced	514 (302,882)	536 (316,930)	537 (312,918)	592 (349,1016)
	THLHP		0 (0,154)	0 (0,196)	0 (0,107)	0 (0,149)
CRP	NHANES	Full	1.48 (0.25,8.08)	4.03 (0.70,23.57)	5.04 (0.87,30.26)	3.47 (0.59,21.19)
	THLHP		1.32 (0.24,7.64)	3.92 (0.60,24.56)	3.91 (0.65,24.68)	1.67 (0.24,9.68)
	NHANES	Reduced	1.82 (0.23,16.77)	4.95 (0.55,37.51)	6.62 (0.86,53.60)	5.54 (0.75,44.26)
	THLHP		1.25 (0.18,9.58)	3.27 (0.40,29.37)	4.30 (0.45,31.41)	1.67 (0.19,15.12)
